# Multiple stochastic pathways in forced peptide-lipid membrane detachment

**DOI:** 10.1038/s41598-018-36528-4

**Published:** 2019-01-24

**Authors:** Milica Utjesanovic, Tina R. Matin, Krishna P. Sigdel, Gavin M. King, Ioan Kosztin

**Affiliations:** 1 Department of Physics and Astronomy, University of Missouri, Columbia, MO 65211 USA; 2 Present Address: Department of Anesthesiology, Weill Cornell Medicine, New York, NY 10065 USA; 3 Present Address: Department of Physics and Astronomy, California State Polytechnic University, Pomona, CA 91768 USA; 4 Department of Biochemistry, University of Missouri, Columbia, MO 65211 USA

## Abstract

We have used high resolution AFM based dynamic force spectroscopy to investigate peptide-lipid membrane interactions by measuring the detachment (last-rupture) force distribution, *P*(*F*), and the corresponding force dependent rupture rate, *k*(*F*), for two different peptides and lipid bilayers. The measured quantities, which differed considerably for different peptides, lipid-membranes, AFM tips (prepared under identical conditions), and retraction speeds of the AFM cantilever, could not be described in terms of the standard theory, according to which detachment occurs along a single pathway, corresponding to a diffusive escape process across a free energy barrier. In particular, the prominent retraction speed dependence of *k*(*F*) was a clear indication that peptide-lipid membrane dissociation occurs stochastically along several detachment pathways. Thereby, we have formulated a general theoretical approach for describing *P*(*F*) and *k*(*F*), by assuming that peptide detachment from lipid membranes occurs, with certain probability, along a few dominant diffusive pathways. This new method was validated through a consistent interpretation of the experimental data. Furthermore, we have found that for moderate retraction speeds at intermediate force values, *k*(*F*) exhibits catch-bond behavior (i.e. decreasing detachment rate with increasing force). According to the proposed model this behavior is due to the stochastic mixing of individual detachment pathways which do not convert or cross during rupture. To our knowledge, such catch-bond mechanism has not been proposed and demonstrated before for a peptide-lipid interaction.

## Introduction

Peptide-lipid interactions are essential for understanding numerous cellular processes and their mechanisms. Both experimental and theoretical study of these interactions is notoriously difficult due to the complexity of the system and the wide range of time and length scales involved. The magnitude of these interactions differs significantly with peptide and lipid species, as well as with solution conditions. A number of methods have been successfully applied to study peptide-lipid interactions^1^. In particular, atomic force microscopy (AFM) based single-molecule force spectroscopy^2–4^ can be used to quantitatively probe the strength and range of peptide-lipid membrane interactions, by measuring the detachment (last rupture) force, *F*, through repeated AFM retraction experiments. The standard approach to interpret the measured histogram, *P*(*F*), of the stochastic force, *F*, is to model the peptide-lipid membrane detachment as a diffusive escape process across a free energy barrier^5–8^. This single detachment pathway model is characterized by (i) an energy parameter (barrier height Δ*U*_0_), (ii) a geometric parameter (attraction range Δ*x*_0_), and (iii) a kinetic parameter (intrinsic escape rate *k*_0_, or escape time *τ*_0_ = 1/*k*_0_). The values of the model parameters are determined by fitting the theoretical prediction to the experimental *P*(*F*). While such an approach is applicable to other force-induced molecular transitions (e.g., unfolding of proteins^9–14^, unzipping of nucleic acid hairpins^15,16^, ligand-receptor dissociation^17,18^, etc.), our experimental results indicate that the single detachment pathway model fails for peptide-lipid membrane systems, most likely due to their complexity. For such systems, *P*(*F*) histograms show noticeable differences even for otherwise identically prepared samples, and depend strongly on other experimental factors, such as the retraction speed, *v*.

In general, whenever the single detachment pathway model is applicable, the force dependent detachment rate, *k*(*F*), which can be obtained directly from the experimental histogram *P*(*F*), is independent of *v* and increases monotonically with *F*^8,19^. Our experiments show that, for peptide-lipid membrane detachment, *k*(*F*) depends strongly on the retraction speed and has non-monotonic behavior in *F*, thus implying that for such systems multiple detachment pathways are in play.

The purpose of this paper is to formulate and apply a general, multi-pathway model for describing the detachment force distribution, *P*(*F*), and the corresponding *k*(*F*), of forced detachment of peptides from lipid membranes. We demonstrate the viability of the proposed model by applying it to AFM retraction experiments, performed under different experimental conditions, that involve two different peptides (of different length) and two species of lipid bilayers. Our model assumes that the detachment process can proceed stochastically along a few, *N* > 1, dominant pathways, with probabilities *w*_*n*_, *n* = 1, …, *N*, such that ${\sum }_{n=1}^{N}{w}_{n}=1$. Similarly to the standard theory^8,19^, each pathway is modeled as a diffusive escape process across a free energy barrier, characterized by the parameters Δ*U*_0*n*_, Δ*x*_0*n*_ and *k*_0*n*_. We identify these pathways as final rupture events involving either one or two residues of the peptide. Indeed, due to its polymeric nature, the separation of the peptide from the membrane during retraction proceeds residue-by-residue. Right before detachment, the peptide-lipid membrane contact is restricted to a single residue, in general, located at the end of the peptide away from the AFM tip. Thus, depending on the time resolution of the AFM instrument, *F* extracted from the retraction force time series, corresponds to the dissociation of the last residue, or the dissociation in rapid succession of the last two (or more) residues still in contact with the membrane. Thus, approximate values of the peptide residue and lipid species specific energetic (Δ*U*_0*n*_) and geometric (Δ*x*_0*n*_) parameters can be obtained from previous free energy profile studies of individual residue analogs interacting with model lipid membranes^20,21^, leaving as effective fitting parameters only the intrinsic detachment rates *k*_0*n*_ and the pathway weight factors *w*_*n*_. Furthermore, once the kinetic parameters (*k*_0*n*_) have also been determined for a specific peptide and lipid-membrane system, by changing experimental conditions (e.g., modifying the retraction speed *v*, or exchanging - otherwise identically prepared - AFM tips) one only needs to tune the weights *w*_*n*_ in order to match the experimental *P*(*F*) and *k*(*F*) with the theoretical model. Indeed, in general, the probability that detachment follows a certain pathway should change by modifying the experimental conditions.

In spite of its formal simplicity, the proposed theoretical model is capable of describing, and interpreting in a consistent manner both *P*(*F*) and *k*(*F*) for all the different AFM retraction experiments reported here. In addition, we find that for intermediate retractions speeds and rupture forces, *k*(*F*) exhibits “catch-bond” behavior^22–24^ when, counter intuitively, the detachment rate decreases with increasing *F*. We show that this behavior is due to properly weighted, force dependent contributions by different detachment pathways to *k*(*F*). Another finding is that the intrinsic (detachment-) off-rate, ${k}_{0}=k(0)={\sum }_{n=1}^{N}{w}_{n}{k}_{0n}$, of the peptide from the membrane (within reasonable numerical errors) is independent of *v*, in spite of the fact that the weights *w*_*n*_ are *v*-dependent while *k*_*n*0_ are not.

It should be noted that, recently, others have also suggested that force-induced transitions in complex molecular systems should involve several reaction pathways, and presented detailed model calculations for different relevant scenarios^25^. However, we are not aware of any study that uses a multiple detachment pathway approach to consistently describe and interpret single molecule dynamic force spectroscopy data, as reported here.

## Theory

**Figure 1:**
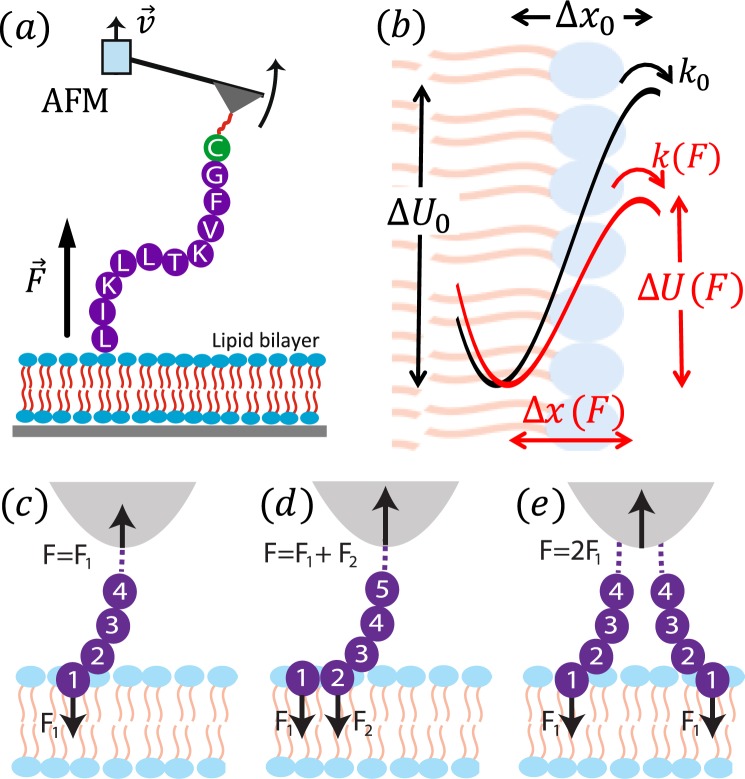
(**a**) Experimental setup showing SecA2-11 interacting with a supported lipid bilayer. (**b**) Physical model. Peptide detachment involves the (**c**) last amino acid (AA) or (**d**) last and next-to-last AA, or occasionally the (**e**) last two AA from neighboring peptide chains.

The forced detachment of a short peptide (biomolecule) from a lipid membrane (substrate), observed in AFM single-molecule dynamic force spectroscopy experiments (Fig. 1a), is a stochastic process that customarily is modeled as a diffusive escape event across a free energy barrier^5–8^. The reaction coordinate, *x*, is defined as the separation between peptide and membrane, along the normal direction to the latter. In the absence of a pulling force, *F*, the intrinsic free energy profile (potential of mean force or PMF), *U*_0_(*x*), is characterized by the separation ${\rm{\Delta }}{x}_{0}={x}_{\cap }-{x}_{\cup }$ between the positions of the equilibrium bound state (${x}_{\cup }$) and transition state (${x}_{\cap }$), and the barrier height (activation energy), Δ*U*_0_, separating these two states (see Fig. 1b). Here we model the PMF by a widely used linear-cubic potential ${U}_{0}(x)={\rm{\Delta }}{U}_{0}[(3/2)(x/{\rm{\Delta }}{x}_{0})-2(x/{\rm{\Delta }}{x}_{0}{)}^{3}$], which was shown to be suitable for studying forced detachment processes^8^. In addition, the dynamics of the detachment process also depends on an effective diffusion coefficient, *D*, or, equivalently, an intrinsic escape rate *k*_0_, or escape time *τ*_0_ = 1/*k*_0_.

In a constant pulling speed (force-ramp) AFM experiment, the applied force, *F*(*t*), modifies the PMF, i.e., *U*(*x*|*F*) = *U*_0_(*x*) − *Fx*, thus facilitating the detachment (last rupture) process by reducing both the barrier height, ${\rm{\Delta }}U(F) < {\rm{\Delta }}{U}_{0}$, and separation, ${\rm{\Delta }}x(F) < {\rm{\Delta }}{x}_{0}$ (Fig. 1b). The stochastic process *x*(*t*) starts at (or about) ${x}_{\cup }$ and terminates with detachment, when it reaches the absorbing boundary at ${x}_{\cap }$. The corresponding detachment force is also a stochastic quantity whose distribution function, *P*(*F*), can be constructed from repeated AFM retraction experiments. Our goal is to determine the model parameters Δ*U*_0_, Δ*x*_0_ and *k*_0_ that reproduce *P*(*F*) obtained from single-molecule AFM force spectroscopy experiments. The theoretical approach for determining *P*(*F*) requires several approximations that are discussed below.

### Detachment along a single pathway

The key quantity for modeling molecular detachment processes along a given pathway is the survival probability (i.e., the probability that bonding is still intact at time *t*) defined as $S(t|z)={\int }_{a}^{b}p(x,t|z)\,dx$, where *p*(*x*, *t*|*z*) is the (transition) probability that the stochastic process *x*(*t*) has the value *x* (with *a* ≤ *x* ≤ *b*) at time *t*, assuming that at *t* = 0 it had the sharp value *z* ≡ *x*(0). In our case $z={x}_{\cup }$, and there is an absorbing boundary at $b={x}_{\cap }$, and a reflective boundary at $a < {x}_{\cup }$. When *U*(*x*) diverges rapidly for $x < {x}_{\cup }$, one can set *a* = −∞.

Because the transition probability obeys the adjoint Smoluchowski equation^26^, $${{\rm{\partial }}}_{t}p(x,t|z)={{\mathscr{L}}}_{S}^{\dagger }(z)\,p(x,t|z)$$, with $${ {\mathcal L} }_{S}^{\dagger }(z)=D\exp [\beta U(z)]{\partial }_{z}$$$$\exp [\,-\,\beta U(z)]{\partial }_{z}=D{\partial }_{z}^{2}-\beta DU^{\prime} (z){\partial }_{z}$$, the equation of motion for the survival probability reads $$\dot{S}(t|z)\equiv {\partial }_{t}S$$$$(t|z)={ {\mathcal L} }_{S}^{\dagger }(z)\,S(t|z)$$, subject to the initial condition *S*(0|*z*) = 1, and boundary condition *S*(*t*|*x*_∩_) = 0. In general, *S*(*t*|*z*) can be calculated only numerically. However, a practical analytical approximation can be obtained by employing the mean-detachment-time (MDT), or mean-first-passage-time (MFPT)^26^ approximation. To this end, note that the distribution function of the detachment time is given by $${\mathscr{P}}(t)=-\,\dot{S}(t|z)$$. Thus, the MDT, $$\tau (z)={\int }_{0}^{\infty }t\,{\mathscr{P}}(t)\,dt={\int }_{0}^{\infty }S(t|z)\,dt$$. Consequently, $${ {\mathcal L} }_{S}^{\dagger }(z)\,\tau (z)={\int }_{0}^{\infty }{ {\mathcal L} }_{S}^{\dagger }(z)\,S(t|z)\,dt={\int }_{0}^{\infty }\dot{S}(t|z)\,dt=-\,1$$. This is the well-known MFPT equation, which can be integrated exactly with the result^26^

1 $$\tau (z)={D}^{-1}{\int }_{z}^{{x}_{\cap }}dx\exp [\beta U(x)]{\int }_{a}^{x}dy\exp [\,-\,\beta U(y)].$$

Clearly, the (mean) detachment time $\tau =\tau ({x}_{\cup })$. Note that in the large barrier limit, Eq. (1) turns into the widely used Kramers’ formula for the escape time^6,8,27,28^.

In general, the MDT (or quasi-adiabatic) approximation is applicable when *τ* is much larger than the characteristic relaxation time of the unruptured system. In single-molecule dynamic force AFM experiments, this approximation usually holds for sufficiently small force loading rates, $\dot{F}=dF/dt$. For simplicity, here we consider only constant loading rates, with $\dot{F}={k}_{s}v$, where *k*_*s*_ is the stiffness of the AFM cantilever and *v* is the constant retraction speed. While the presence of the linker between the tip of the cantilever and biomolecule leads to a weak force dependence of $\dot{F}$, in many cases this dependence can be either neglected or accounted for through a mean spring constant ${k}_{s}^{\ast }\sim {k}_{s}$.

In the MDT approximation, the equation of motion for the survival probability is simplified, $\dot{S}(t)={ {\mathcal L} }_{S}^{\dagger }S(t)\approx -\,{\tau }^{-1}S(t)=-\,kS(t)$, and it can be integrated with the result $S(t)=\exp (\,-\,{\int }_{0}^{t}kdt^{\prime} )$. Finally, in terms of the survival probability, the rupture force distribution is $P(F)=-\,dS/dF=-\,\dot{S}/\dot{F}$, which in the MDT approximation becomes

2 $$P(F)=\frac{k(F)}{\dot{F}}S(F)=\frac{k(F)}{\dot{F}}\exp \,[-\,{\int }_{0}^{F}\frac{k(f)}{\dot{F}}df],$$

where the force dependent detachment rate *k*(*F*) = 1/*τ*(*x*_∪_), with the MDT given by Eq. (1). After little algebra, *k*(*F*) can be expressed from Eq. (2) only in terms of the experimental histogram *P*(*F*) and force loading rate, i.e.,

3 $$k(F)=\frac{\dot{F}P(F)}{1-D(F)},\,D(F)={\int }_{0}^{F}P(f)df.$$

Either of the last two equations can be used to determine the sought model parameters Δ*U*_0_, Δ*x*_0_ and *k*_0_, by fitting the theoretical prediction to the experimental data. However, Eq. (3) is crucial in determining whether the detachment process follows a single pathway or multiple pathways. Indeed, in the case of a single detachment pathway, *k*(*F*) is independent of the retraction speed *v*. Thus, *k*(*F*|*v*) obtained from AFM experiments using different retraction speeds must collapse to the same, monotonically increasing curve^8^. On the other hand, whenever *k*(*F*|*v*) shows significant *v* dependence (and possibly non-monotonic behavior in *F*) it is a clear indication that the detachment process occurs stochastically along several different pathways.

### Detachment along multiple pathways

It is natural to assume that the detachment of a peptide from a lipid membrane, due to the complex, stochastic nature of their interaction, may follow a number of different, independent pathways. In such case, the probability that detachment has not occurred yet at time *t* (survival probability) can be written as a weighted sum $S(t)={\sum }_{n=1}^{N}{w}_{n}{S}_{n}(t)$, where *w*_*n*_ is the probability of detachment along the *n*-th pathway. Clearly, ${\sum }_{n=1}^{N}{w}_{n}=1$. Each detachment pathway is characterized by the parameters Δ*U*_0*n*_, Δ*x*_0*n*_ and *k*_0*n*_, *n* = 1, …, *N*. Using the MDT approximation described in the previous section, it is straightforward to show that, in the case of multiple pathways, the detachment force distribution can be written

4a $$P(F)=-\,\frac{dS}{dF}=\sum _{n=1}^{N}{w}_{n}{P}_{n}(F),$$

where for the *n*-th pathway

4b $${P}_{n}(F)=\frac{{k}_{n}(F)}{\dot{F}}{S}_{n}(F),$$

with

4c $${S}_{n}(F)=\exp (-\,{\int }_{0}^{F}\frac{{k}_{n}(f)}{\dot{F}}df).$$

Similarly to Eq. (1), the force dependent detachment rate for the *n*-th pathway

5 $${k}_{n}(F)=D{({\int }_{{x}_{n\cup }}^{{x}_{n\cap }}dx\exp [\beta {U}_{n}(x)]{\int }_{-\infty }^{x}dy\exp [-\beta {U}_{n}(y)])}^{-1},$$

and the corresponding intrinsic detachment rate *k*_0*n*_ = *k*_*n*_(0). Equations (4), together with Eq. (5), are the multiple pathways equivalent of Eq. (2), and can be used to determine the model parameters Δ*U*_0*n*_, Δ*x*_0*n*_ and *k*_0*n*_, along with the weight factors *w*_*n*_, by fitting the experimental data.

Finally, the mean, force dependent, detachment rate for multiple pathways is given formally by the same Eq. (3) as for a single detachment pathway, except that for *P*(*F*) one needs to use Eq. (4). Thus

6 $$k(F)=\sum _{n=1}^{N}{\alpha }_{n}(F){k}_{n}(F),\,{\alpha }_{n}(F)=\frac{{w}_{n}{S}_{n}(F)}{\sum _{n=1}^{N}{w}_{n}{S}_{n}(F)}.$$

Depending on the pathways involved, due to the force dependence of the *α*_*n*_(*F*) coefficients, *k*(*F*) may depend strongly on the retraction speed, *v*, and may also exhibit non-monotonic dependence (i.e., *catch-bond* behavior^22^) on the detachment force, *F*.

### Double and multiple detachment events

In many cases, right before detachment, the contact between a peptide and lipid membrane is confined to a single amino acid (Fig. 1c), and the experimentally recorded detachment (last rupture) force corresponds to such situation. However, due to the limited time resolution of the AFM or other experimental complications, occasionally the recorded detachment force corresponds to a double rupture event, e.g., when either the last two amino acids of a single peptide (Fig. 1d), or the last amino acids of two copies of the peptide (attached to the tip of the AFM cantilever; Fig. 1e), in contact with the membrane, rupture in rapid succession. In double rupture cases, the measured detachment force is roughly twice as big as for the corresponding single detachment event. Thus, double rupture events can be regarded as independent detachment pathways with a weight factor ${w}_{m}^{\mathrm{(2)}}$ and a rupture force distribution ${P}_{m}^{\mathrm{(2)}}(F)=\mathrm{(1/2)}{P}_{m}(F\mathrm{/2)}$, where ${P}_{m}(F)\equiv {P}_{m}^{\mathrm{(1)}}(F)$ corresponds to single residue detachment. In principle, one may consider multiple detachment events that involve the rupture in rapid succession (that cannot be time-resolved experimentally) of *p* > 2 amino acids, which can be modeled as pathways with ${w}_{n}^{(p)}$ and ${P}_{m}^{(p)}(F)=(1/p){P}_{m}(F/p)$. However, the occurrence of multiple detachment events with *p* > 2 is much smaller than those involving single and double ruptures, and usually can be neglected.

## AFM Experiments

Following our group’s recent work, high precision AFM-based single molecule force spectroscopy experiments were performed to measure the detachment (last rupture) force between two different peptides and two lipid bilayer species^2^. Peptides were derived from SecA, which is a large (901 amino acid) peripheral membrane protein central to the general Sec system. We focused on two distinct segments of SecA: the extreme N-terminal 10 amino acids (SecA2-11) as well as a 20 amino acid segment in the middle of the protein (SecA600-619). The membrane bilayer was varied between 1-palmitoyl-2-oleoyl-sn-glycero-3-phosphocholine (POPC) which is a model zwitterionic lipid and *E. coli* polar lipid which is a mixture of zwitterionic (phosphatidylethanolamine) and charged (phosphatidylglycerol and cardiolipin) lipids (all lipids were purchased from Avanti).

Peptides were synthesized in house using solid-phase synthesis on Sieber amide resin and standard Fmoc/tBu chemistry for linear elongation resulting in purities ≥95% with the follow sequences: SecA600-619: SDRVSGMMRKLGMKPGEAIE-C and SecA2-11: LIKLLTKVFG-C. The cysteine residue at the C-terminus of both peptides allowed site-specific, covalent functionalization onto AFM tips via a 9.5 nm long PEG linker. Incubation conditions were optimized to yield ~1 peptide tethered at the tip apex, as described previously^2,29^. To allow pN level precision, we utilized bioloever long cantilevers (Olympus) without metallic coatings^30^. Cantilever spring constants (*k*_*s*_) were in the range of 3–8 pN/nm, determined via the thermal calibration. Supported bilayers were formed by vesicle fusion to clean glass surfaces^31^ which were rinsed (0.1 mL buffer solution, 3x) prior to force spectroscopy experiments. Such conditions result in uniform bilayer coverage over large areas^2,31^.

Force spectroscopy experiments were carried out in aqueous buffer solution (10 mM Hepes pH 7.6, 300 mM KAc, 5 mM Mg(Ac)_2_) at ~30° using a commercial AFM (Cypher, Asylum Research). The speed, *v*, was controlled by the piezoelectric stage affixed to the base of the cantilever. Rupture forces >60 pN were rare and excluded from analysis. Additionally, rupture events occurring <3 nm above the lipid surface were excluded (to minimize non-specific interactions). Unless otherwise specified, for each experimental condition, data from ≥5 distinct tips were aggregated, yielding between 275–529 distinct rupture events per condition.

## Results and Discussion

In a series of high precision AFM-based single molecule force spectroscopy experiments we have measured repeatedly, under different experimental conditions, the (stochastic) peptide-lipid membrane detachment force, *F*, using two different peptides (SecA2-11 and SecA600-619) and two lipid (zwitterionic POPC and charged *E. coli* polar) membranes. The constructed *P*(*F*) histograms showed significant variation from experiment to experiment, even for the same peptide-lipid membrane system. Attempts to model *P*(*F*) by using the theory for a single detachment pathway failed. We attribute this failure to the complexity of the peptide-lipid membrane interactions. Here we show that in fact *P*(*F*) can be modeled in a consistent way by assuming that peptide-lipid membrane detachment involves a small number (usually *N* = 3 or 4) of dominant pathways of single and double rupture events. The modeling strategy is as follows. First, one identifies the residues at the end of the peptide that are most likely to rupture last. For SecA2-11 (SecA600-619) these residues are L and I (S, D, and R). Next, the corresponding values of the PMF parameters, Δ*U*_0*n*_ and Δ*x*_0*n*_, are identified from previous MD simulation studies^20,21^, which reconstructed the PMF of various residues interacting with POPC bilayers. These values are listed in Table 1. Finally, the intrinsic detachment rates *k*_0*n*_ (kinetic parameters) and occurrence probabilities *w*_*n*_ (weight coefficients) for each participating detachment pathway (*n* = 1, …, *N*) are determined by fitting the experimental *P*(*F*) histograms using Eqs (4) and (5). It should be emphasized that the PMF parameters (Δ*U*_0*n*_ and Δ*x*_0*n*_) are determined by the nature of the peptide and lipid membrane and, thus, their values should not be changed during the fitting process. Also, one expects that the rates *k*_0*n*_ may depend slightly on the experimental conditions (e.g., retraction speed *v*, AFM cantilever tip), but not as much as the weight factors *w*_*n*_, which may change considerably from experiment to experiment. While the convergence of *w*_*n*_ to a peptide-lipid membrane specific value may require a very large number of measurements, the parameters identifying the individual pathways (i.e., Δ*U*_0*n*_, Δ*x*_0*n*_, and *k*_0*n*_) should be obtainable from a relatively small number of single molecule experiments.

**Table 1:** PMF model parameters describing the interaction of selected residues with POPC lipid bilayer^20,21^.

Residue	Δ*U*_0_ [*k*_*B*_*T*]	Δ*x*_*o*_ [nm]
L	8.0	1.0
I	10.0	1.3
S	2.0	0.7
R	8.2	1.3

Next, we consider three exemplary cases to demonstrate the viability of the method described above for interpreting detachment force histograms for peptide-lipid membrane systems.

### *P*(*F*): AFM-tip dependence

**Figure 2:**
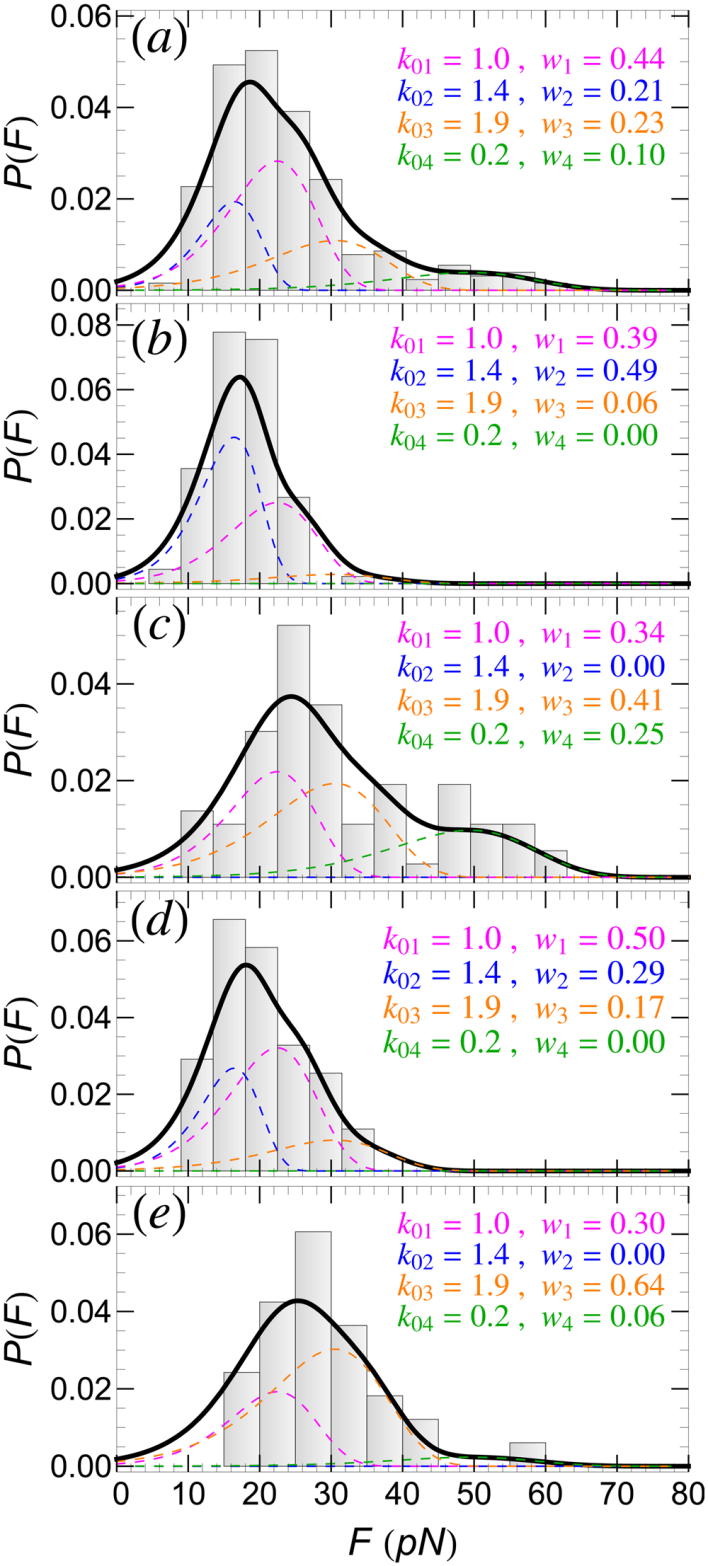
Detachment force histogram, *P*(*F*), of SecA2-11 interacting with POPC bilayer using data from (**a**) all AFM tips, and (**b**–**e**) four individual AFM tips. In each case, *P*(*F*) can be fitted well by using four detachment pathways (solid-thick curves). Contributions to *P*(*F*) from the *n*-th (*n* = 1, ..., 4) detachment pathways are shown as colored dashed curves; the corresponding intrinsic rupture rates, *k*_0*n*_, and weights, *w*_*n*_, are also listed.

The experimental *P*(*F*) histogram of SecA2-11 interacting with POPC lipid bilayer (for *v* = 100 nm/s) shown in Fig. 2a can be fitted well by assuming *N* = 4 detachment pathways. The quality of this fit, as well as the other *P*(*F*) fits reported in this paper, is rather good, characterized by a coefficient of determination *R*^2^ > 0.9. Two of these (*n* = 1, 2) are identified as single ruptures involving the last two residues, L and I, of the peptide. The other two pathways (*n* = 3, 4) correspond to double rupture processes involving the same residues. The corresponding PMF parameters are listed in Table 1, while the fitting parameters, *k*_0*n*_ and *w*_*n*_, are listed in Fig. 2a. Note that, in this case, the single rupture events (65%) are more prevalent than double ruptures (33%). Also, the predominant four pathways account for 98% of *P*(*F*); the remaining 2% correspond to other pathways that occur only seldom and, thus, can be neglected.

The *P*(*F*) histogram in Fig. 2a contains data from several AFM retraction experiments, performed with different, but otherwise identically prepared, AFM tips. Reflecting the stochastic nature of single molecule experiments, the *P*(*F*) histograms for individual tips, shown in Fig. 2b–e, have completely different shapes. However, all these different histograms can be well fitted assuming the same *N* = 4 detachment pathways identified above, but with different weight factors *w*_*n*_. This means that, in otherwise identically prepared samples, certain detachment pathways may be favored against others. While, in general, one cannot predict the prevalence of a particular pathway in individual experiments, it is still remarkable that *P*(*F*) can be reproduced by only using *w*_*n*_’s as actual fitting parameters. Thus, the detachment pathways, identified through three parameters (i.e., Δ*U*_0*n*_, Δ*x*_0*n*_, and *k*_0*n*_), can be regarded as fingerprints of a specific peptide-lipid membrane system.

### *P*(*F*): retraction speed, *v*, dependence

**Figure 3:**
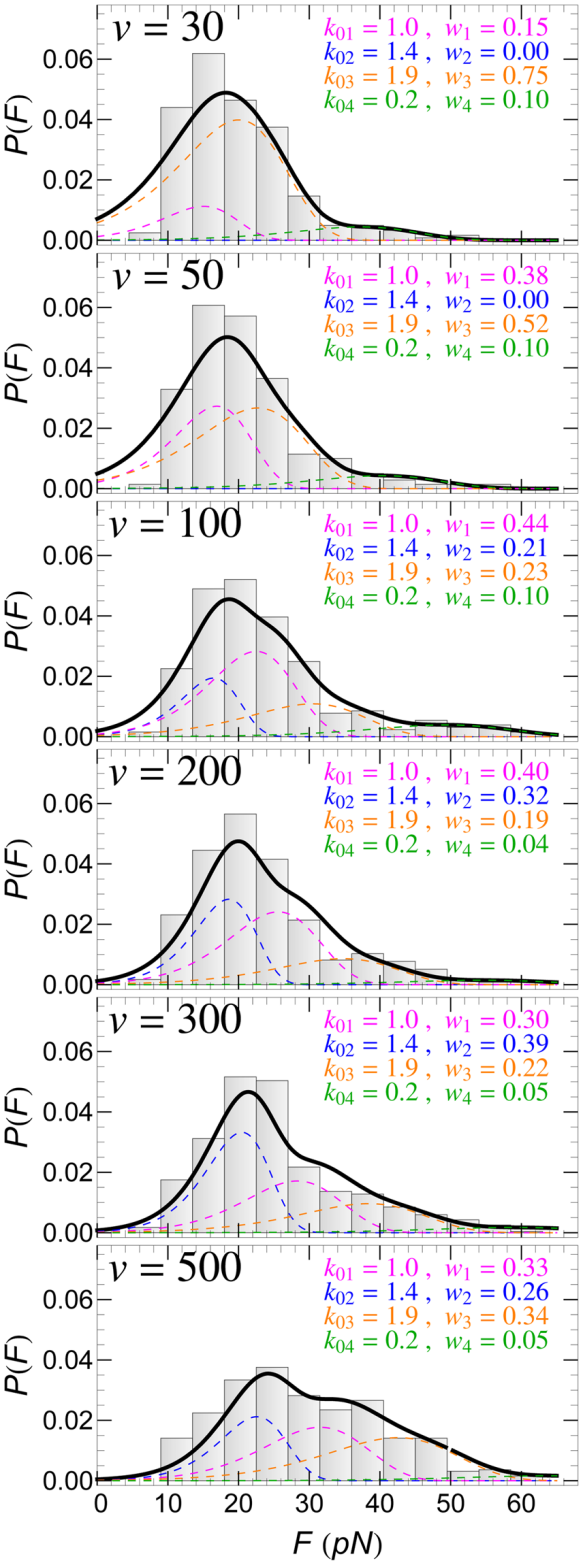
Detachment force histogram, *P*(*F*), of SecA2-11 interacting with POPC bilayer for different retraction speeds (in nm/s) of the AFM tip. In each case, *P*(*F*) can be fitted well by using four detachment pathways (solid-thick curves). Contributions to *P*(*F*) from the *n*-th (*n* = 1, ..., 4) detachment pathways are shown as colored dashed curves; the corresponding intrinsic rupture rates, *k*_0*n*_, and weights, *w*_*n*_, are also listed.

The experimental *P*(*F*) histogram of SecA2-11 interacting with POPC lipid bilayer for six different retractions speeds are shown in Fig. 3. In all these cases too, *P*(*F*) can be fitted well by assuming the same four detachment pathways identified in the previous section, and by simply adjusting the corresponding weight factors *w*_*n*_. It appears that for the lowest retraction speeds, *v* = 30 and 50 nm/s, the double rupture pathways are the dominant ones, accounting respectively for 85% and 62% of *P*(*F*). For higher speeds, the situation is reversed, in favor of the single rupture pathways.

**Figure 4:**
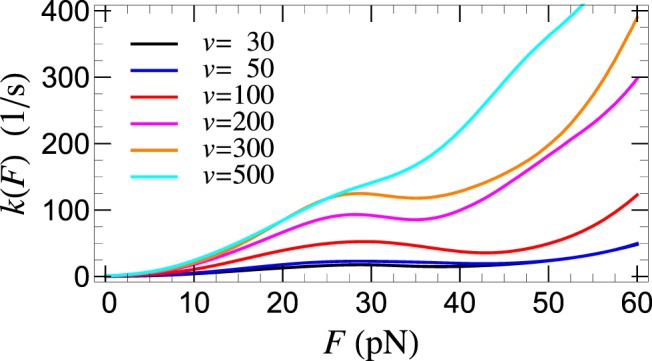
Force dependent detachment rate, *k*(*F*), determined from Eq. (3), for SecA2-11 interacting with POPC bilayer, for the listed retraction speeds (in nm/s) of the AFM tip.

**Figure 5:**
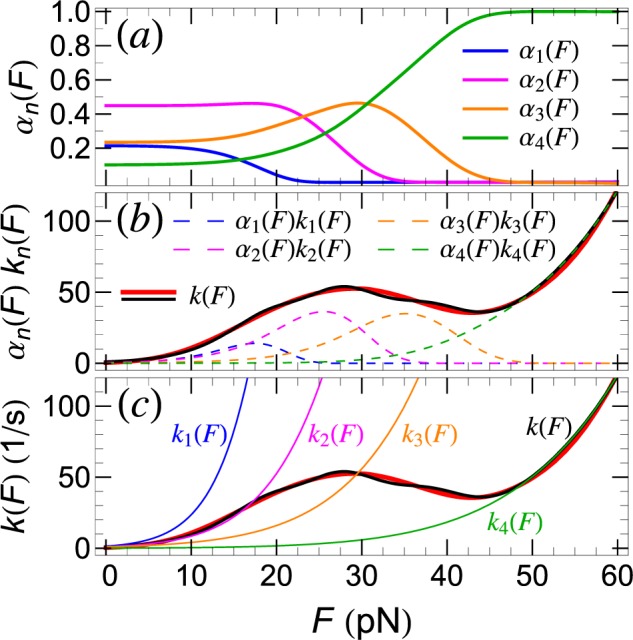
Force dependent detachment rate, *k*(*F*), of SecA2-11 from POPC lipid bilayer obtained from AFM retraction experiments with *v* = 100 nm/s retraction speed. (**a**) Force dependent weight factors for each detachment pathway. (**b**) *k*(*F*) derived using *P*(*F*) from experiment (thick, solid red curve) and our theoretical modeling (solid black curve). The weighted contributions of individual pathways to *k*(*F*), i.e., *α*_*n*_(*F*)*k*_*n*_(*F*), *n* = 1, …, 4, are also shown (dashed curves). (**c**) Pathway specific, force dependent rupture rates, *k*_*n*_(*F*). For reference, the experimental and theoretical *k*(*F*) are also shown as in (**b**).

As already mentioned, the strong *v* dependence of *k*(*F*), determined from the experimental *P*(*F*) by means of Eq. (3), and shown in Fig. 4, clearly demonstrates that peptide-lipid bilayer detachment cannot be described by a single detachment pathway, thus lending support to our multiple pathways method. While the individual rates, *k*_*n*_(*F*), for each detachment pathway are monotonically increasing with *F*, the non-monotonic behavior of *k*(*F*), given by Eq. (6), is due to the *F*-dependent contributions [through the coefficients *α*_*n*_(*F*)] of the individual pathways. The situation is illustrated, for *v* = 100 nm/s, in Fig. 5.

It is remarkable that, for intermediate forces, the stochastic mixing between the different pathways may lead to “*catch-bond*” behavior^22–24^, where counter-intuitively the detachment rate decreases with the increase of the applied force (Fig. 5b). To the best of our knowledge, this is the first time when a catch-bond mechanism based on detachment pathway mixing has been proposed and demonstrated for a peptide-lipid membrane interaction. It should be emphasized that the origin of the catch-bond in this case is quite different from the so-called (phenomenological) “*two-pathway*” model, according to which catch-bond behavior in ligand-receptor systems comes about through pathway switching (*inner conversion*)^24^. Here, individual peptide-lipid detachment events occur, with a given probability, along well defined pathways, which do not cross or convert during rupture. Thus, the origin of the catch-bond behavior in peptide-lipid membrane interaction is due to the experimental inseparability of the multiple pathways along which detachment may occur. While the contribution of individual pathways to *P*(*F*) are force independent (*w*_*n*_), the weight of the same contribution (*α*_*n*_) to the effective detachment rate *k*(*F*) decreases with force because fewer attachments are still intact (see also Fig. 5a and Eq. (6)). For sufficiently large forces, when only two pathways contribute to *k*(*F*), the latter should exhibit, in a narrow force range, catch-bond behavior with *dk*/*dF* < 0 (see Fig. 5).

**Figure 6:**
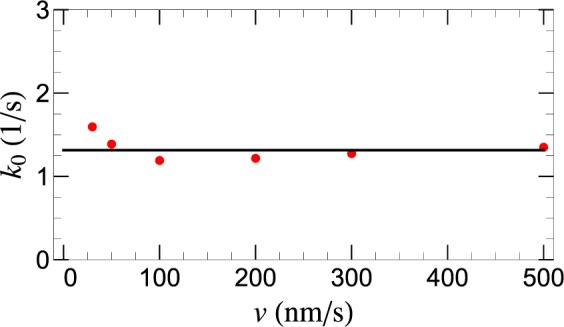
AFM retraction speed, *v*, dependence of the intrinsic detachment rate, *k*_0_ = *k*(*F* = 0), (red filled-circles) of SecA2-11 interacting with POPC bilayer. Horizontal black line represents the mean value 〈*k*_0_〉.

Furthermore, it is also remarkable that, in spite of the significant *v*-dependence of *P*(*F*) and *k*(*F*), the intrinsic (detachment) off-rate, ${k}_{0}=k(0)={\sum }_{n=1}^{4}{w}_{n}{k}_{0n}\approx 1.2\,{{\rm{s}}}^{-1}$, obtained from Eq. (6) for *F* = 0, appears to be independent of *v*, as shown in Fig. 6. This, normally expected but non-trivial result, lends further support to our theoretical model.

### *P*(*F*): peptide and membrane dependence

Finally, we demonstrate that the proposed multiple detachment pathways method works equally well for other peptides and types of lipid bilayers.

**Figure 7:**
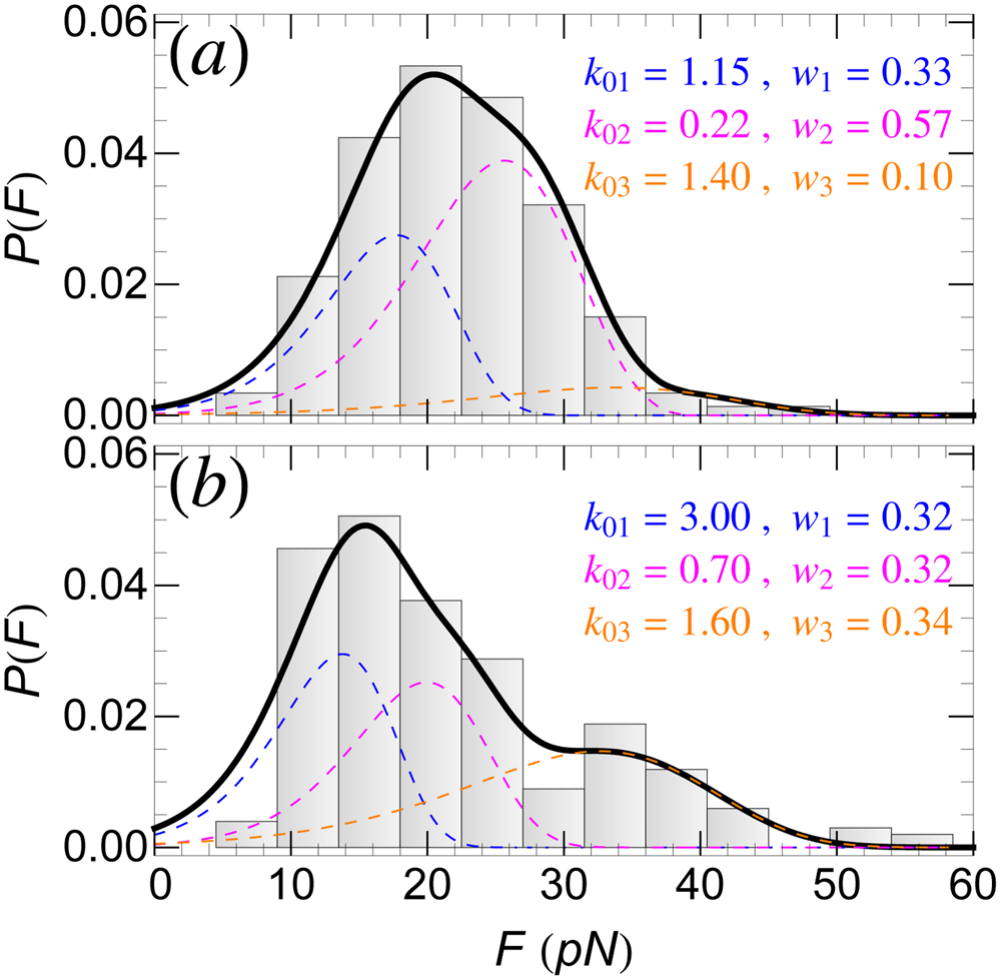
Detachment force histogram, *P*(*F*), of SecA600-619 peptide interacting with (**a**) POPC bilayer, and (**b**) *E. coli* membrane. *P*(*F*) can be fitted well by using three detachment pathways (solid-thick curves). Contributions to *P*(*F*) from the *n*-th (*n* = 1, ..., 3) detachment pathways are shown as colored dashed curves; the corresponding intrinsic rupture rates, *k*_0*n*_, and weights, *w*_*n*_, are also listed.

First, consider the detachment of SecA600-619 interacting with POPC (for the same *v* = 100 nm/s as for SecA2-11). The last three residues of this peptide are S, D and R. Previous MD simulation studies^20,21^ have shown that there is essentially no escape barrier for D interacting with POPC, and the one for S is negligibly small (see Table 1). Therefore, in order to fit the experimental *P*(*F*) histogram, one needs to consider two single rupture pathways, associated with residue R (with two different intrinsic rates, *k*_01_ and *k*_02_), along with a third, double rupture pathway that involves two R residues. Similarly to SecA2-11, as shown in Fig. 7a, the experimental *P*(*F*) for SecA600-619 can be fitted well with the theoretical model involving the above three pathways. The values of the fitting parameters, *k*_0*n*_ and *w*_*n*_, are listed in the same figure.

As shown in Fig. 7b, the experimental *P*(*F*) for SecA600-619 interacting with an *E. coli* membrane deviates significantly from the one with POPC bilayer (Fig. 7a). However, in this case too, the same three detachment pathways as for POPC can be used to fit *P*(*F*), by keeping the same (residue specific) PMF parameters but, re-evaluating (as fitting parameters) both the intrinsic kinetic rates *k*_0*n*_ and the weight factors *w*_*n*_. These new values are listed in Fig. 7b. The justification for keeping the same Δ*U*_0*n*_ and Δ*x*_0*n*_ for *E. coli* as for POPC is twofold: (i) the lack of PMF data for peptide residues in *E. coli* membrane, and (ii) the fact that in *E. coli* membrane the zwitterionic lipids are more abundant (~70%) than the anionic ones (~30%). It appears, however, that the presence of charged lipids in the *E. coli* membrane leads to a considerable increase of both the rates *k*_0*n*_, and the frequency of double rupture detachment events at the expense of single ruptures.

## Conclusions

We have used high resolution AFM-based dynamic force spectroscopy to investigate quantitatively peptide-lipid membrane interactions by measuring the detachment (last-rupture) force distribution, *P*(*F*), and the corresponding force dependent rupture rate, *k*(*F*), for two different peptides (SecA2-11 and SecA600-619) and lipid bilayers (zwitterionic POPC and charged *E. coli* polar membrane). The measured quantities, which differed considerably for different peptides, lipid-membranes, AFM tips (prepared under identical conditions), and retraction speeds of the AFM cantilever, could not be described in terms of the standard theory that assumes that detachment occurs along a single pathway. In particular, the prominent *v*-dependence of *k*(*F*) was a clear indication that peptide-lipid membrane detachment occurs stochastically along several detachment pathways.

A main result of this work is the formulation and validation of a general theoretical approach that provides a quantitative description of the detachment force distribution of a peptide from a lipid membrane. The proposed model assumes that detachment occurs, with certain probability (*w*_*n*_), along a few dominant diffusive pathways, characterized by three parameters (Δ*U*_0*n*_, Δ*x*_0*n*_, and *k*_0*n*_). We have identified these pathways with last-rupture events involving one or two residues, in general, located at the end of the peptide. The values of the energetic (Δ*U*_0*n*_) and geometric (Δ*x*_0*n*_) parameters, which are residue and lipid species specific, were derived from existing free energy profile studies, while the kinetic parameters (*k*_0*n*_) and pathway weights (*w*_*n*_) were used as fitting parameters. This new theoretical approach allowed for a consistent interpretation of all our experimental data. Interestingly, even for the same peptide-lipid membrane system, the occurrence frequency of different dominant detachment pathways (measured through *w*_*n*_) showed significant AFM tip dependence, although these tips were prepared identically. However, even if one cannot predict the occurrence probability of a particular pathway in individual experiments, it is quite remarkable that, once the pathways have been identified and characterized, the rupture force histogram *P*(*F*) can be matched by only using *w*_*n*_’s as fitting parameters.

Our theoretical model also reproduced accurately both retraction speed and non-monotonic force dependence of the rupture rate *k*(*F*). For moderate *v* and intermediate *F* values, *k*(*F*) exhibited *catch-bond* behavior, namely a decrease with increasing *F*, before continuing to increase again, as normally expected. According to our model, the origin of the catch-bond mechanism in peptide-lipid membrane interactions is the stochastic mixing of individual detachment pathways, which do not convert or cross during rupture. This catch-bond mechanism is manifestly different from the commonly used “two-pathway” model^24^ for ligand-receptor systems.

Using our theoretical model, we also calculated the intrinsic detachment (or off) rate, $k\mathrm{(0})={\sum }_{n=1}^{N}{w}_{n}{k}_{0n}$, of the peptide SecA2-11 from the lipid bilayer POPC. As expected, the result was independent (within margin of errors) of the retraction speed, which is quite remarkable if we take into account that the pathway weights *w*_*n*_ are *v*-dependent while the individual rates *k*_0*n*_ are not.

To conclude, we notice that, beside the detachment (last rupture) forces, the retraction force time series, *F*(*t*), contains detailed information about the entire peptide-lipid membrane interaction process, including intermediate rupture events. In the framework of our theoretical approach, the latter may be regarded as detachment events of intermediate residues. However, further work is needed to apply and test our theory for such intermediate rupture events.
